# Supplementary material for: APOΕ4 lowers energy expenditure in females and impairs glucose oxidation by increasing flux through aerobic glycolysis
Source: Mol Neurodegener. 2021 Sep 6;16:62. doi: 10.1186/s13024-021-00483-y (PMC8420022; doi:10.1186/s13024-021-00483-y)
Supplement: Supplementary file 2 — Additional file 2: Supplemental Table 1. Top 25 genes classifying the eight distinct astrocyte clusters identified in scRNAseq analysis of E3 and E4 mouse brains (clusters visualized in Fig. 1e). Supplemental Table 2. Age, sex, and APOE genotype of cognitively normal individuals according to Ε4 carriage and age cohort (young = 18–39, middle-aged = 40–65). Values represent means +/− (SD). Supplemental Table 3. Clinical characteristics of cognitively unimpaired individuals according to Ε4 carriage and age cohort (young = 18–39, middle-aged = 40–65). Values represent means +/− (SD). Ca, Caucasian; AA, African American; His, Hispanic; A, Asian; BMI, body mass index. Supplemental Table 4. Plasma metabolites of study participants analyzed by gas chromatography – before and after a dietary glucose challenge. Supplemental Table 5. Pre-screening checklist. A response of “yes” to any of the following resulted in exclusion from the study. Supplemental Fig. 1. Cluster cell counts. Number of cells in each graph-based cluster from all cells (a), and astrocytes only (b). Bars represent mean number of cells in each cluster, with the number of E3 cells (circles) and E4 cells (squares) noted by symbols. Supplemental Fig. 2. APOE expression in single-cells and specific astrocyte clusters. (a) UMAP visualization of E3 (left) and E4 (right) cells showing expression of APOE. APOE expression is primarily limited to cells identified as astrocytes. (b) Expression of APOE in astrocyte-only UMAP (Aldoc + cells). Inset shows the 8 distinct astrocyte clusters. (c) Violin plots showing expression of APOE in all astrocytes (left) and within each astrocyte cluster (right). (**P < 0.01, ***P < 0.001, unpaired t-test, two-tailed). Supplemental Fig. 3. E4 is associated with decreases in many genes of the oxidative phosphorylation KEGG pathway. Pathway map for KEGG pathway “Oxidative Phosphorylation” showing genes differentially expressed between E3 and E4 astrocytes. Genes highlighted in green are do [file 13024_2021_483_MOESM2_ESM.docx]

**Supplemental Material**

| **Top features** | **Cluster 1** | **Cluster 2** | **Cluster 3** | **Cluster 4** | **Cluster 5** | **Cluster 6** | **Cluster 7** | **Cluster 8** |
| --- | --- | --- | --- | --- | --- | --- | --- | --- |
| **1** | *Agt* | *Lhx2* | *Dclk1* | *Agt* | *Gnao1* | *6330403K07Rik* | *Gm28437* | *Gm10925* |
| **2** | *Sparc* | *Pantr1* | *Cspg5* | *Sparc* | *Fam13c* | *Fxyd6* | *CT010467.1* | *Gm28661* |
| **3** | *lgsf1* | *Rgcc* | *Mfge8* | *Lrig1* | *Clmn* | *Meg3* | *Gm10800* | *Gm28437* |
| **4** | *Slc6a11* | *Bmyc* | *Gnao1* | *Trpm3* | *Cadm1* | *Slc38a1* | *Atp1a2* | *Kif5a* |
| **5** | *ltih3* | *Hopx* | *Atp1b1* | *Chl1* | *Hapln1* | *Gfap* | *Ddx17* | *Gm29216* |
| **6** | *A330076C08Rik* | *Ppp1r3g* | *Fjx1* | *Plce1* | *Dclk1* | *Basp1* | *Lars2* | *Bcas1* |
| **7** | *Cd81* | *Cbr3* | *Pde10a* | *A330076C08Rik* | *Chrdl1* | *Bex4* | *Col11a2* | *Gm13339* |
| **8** | *Nkx6-2* | *Gnao1* | *Slco1c1* | *Slc6a11* | *Dio2* | *Rtn1* | *Gm26917* | *Fth1* |
| **9** | *Sfrp5* | *Ddah1* | *Chst2* | *lgsf1* | *Slco1c1* | *Slc6a6* | *Nsmf* | *Mobp* |
| **10** | *Etnppl* | *Ezr* | *Cadm1* | *ltih3* | *Chst2* | *Bex1* | *Rapgef3* | *Rapgef4* |
| **11** | *Cit* | *Chst2* | *Lhx2* | *Spon1* | *Mertk* | *Ndn* | *Gm13339* | *CT010467.1* |
| **12** | *Rdh5* | *ld4* | *Hopx* | *Gm42583* | *Atp1b1* | *App* | *Fgfr3* | *Mbp* |
| **13** | *Nkain4* | *Ptn* | *Gria2* | *Ptch1* | *St6galnac5* | *Map1b* | *Gm21738* | *Rplp1* |
| **14** | *lrx5* | *Atp1b1* | *Pantr1* | *Gria1* | *Ezr* | *Tmsb10* | *Grin2c* | *Kif5c* |
| **15** | *Plce1* | *Crip2* | *Sat1* | *Kcnd2* | *Frmd4a* | *Vim* | *Syne1* | *Nrsn1* |
| **16** | *Spon1* | *Fjx1* | *Kcnk1* | *Lgi1* | *Lhx2* | *Stmn3* | *Fam193b* | *Gng13* |
| **17** | *Cd38* | *Rbp1* | *Fam171b* | *ltpkb* | *Lrrc8c* | *Nsg2* | *Sned1* | *Gm28439* |
| **18** | *Marcks* | *Fam171b* | *St6galnac5* | *Etnppl* | *Grm3* | *Marcksl1* | *Xist* | *Gm20594* |
| **19** | *Gria1* | *Lix1* | *Ppp1r3g* | *Cd38* | *4930488L21Rik* | *Sept3* | *Macf1* | *Rps29* |
| **20** | *Gm42899* | *Ugp2* | *Mgll* | *5031439G07Rik* | *Swap70* | *Pink1* | *Gm10719* | *Rims1* |
| **21** | *Paqr6* | *Mgll* | *Ezr* | *Slc4a4* | *Gria2* | *Pcsk1n* | *Fads2* | *Rps21* |
| **22** | *Mgat4c* | *Slc9a3r1* | *Oaf* | *Fry* | *Pde10a* | *lgfbp5* | *Agrn* | *Rph3a* |
| **23** | *Ptch1* | *Hes5* | *Pmp22* | *Erbb4* | *Fam171b* | *Thbs4* | *Grik5* | *Scrt1* |
| **24** | *Mpp6* | *Cspg5* | *Rgcc* | *Ntrk3* | *Pou3f3* | *Atp1a1* | *C4b* | *Rps19* |
| **25** | *Apoc1* | *Naaa* | *Bmyc* | *Sfrp5* | *Gm44895* | *Pafah1b3* | *Plxnb1* | *Nrgn* |

**Supplemental Table 1.**  Top 25 genes classifying the eight distinct astrocyte clusters identified in scRNAseq analysis of E3 and E4 mouse brains (clusters visualized in Fig. 1e)

|  | Age | | Sex (N) | | *APOE* Genotype (N) | | | | |
| --- | --- | --- | --- | --- | --- | --- | --- | --- | --- |
|  | Average Age (SD) | P | Male | Female | APOE 2/3 | APOE 3/3 | APOE 2/4 | APOE 3/4 | APOE 4/4 |
| Young | | | | | | | | | |
| Ε4 Non-carriers (E4-) | 24.91  (4.84) | 0.486 | 13 | 33 | 8 | 38 | 0 | 0 | 0 |
| Ε4 Carriers (E4+) | 25.84  (6.08) |  | 5 | 20 | 0 | 0 | 2 | 21 | 2 |
| Middle-age | | | | | | | | | |
| Ε4 Non-carriers (E4-) | 51.18  (7.79) | 0.233 | 4 | 11 | 2 | 13 | 0 | 0 | 0 |
| Ε4 Carriers (E4+) | 55.5  (8.78) |  | 1 | 7 | 0 | 0 | 0 | 7 | 1 |

**Supplemental Table 2.**  Age, sex, and APOE genotype of cognitively normal individuals according to Ε4 carriage and age cohort (young=18-39, middle-aged=40-65). Values represent means +/- (SD).

|  | Race (N) | | | | Vitals | | | | | | | | | |
| --- | --- | --- | --- | --- | --- | --- | --- | --- | --- | --- | --- | --- | --- | --- |
|  | Ca | AA | His | A | BMI (kg/m^2^) | P | Waist/  Hip Ratio | P | Systolic (mmHg) | P | Diastolic (mmHg) | P | Lean Body Mass (kg) | P |
| Young | | | | | | | | | | | | | | |
| E4- | 34 | 4 | 3 | 4 | 25.15  (3.83) | 0.93 | 0.86  (0.1) | 0.56 | 123.2  (21.2) | 0.79 | 72.3  (14.4) | 0.9 | 48.2  (7.3) | 0.33 |
| E4+ | 22 | 3 | 0 | 0 | 25.06  (4.72) |  | 0.87  (0.1) |  | 121.8  (18.5) |  | 72.1  (12.1) |  | 46.4  (7.2) |  |
| Middle-age | | | | | | | | | | | | | | |
| E4- | 12 | 3 | 1 | 0 | 25.83  (6.07) | 0.45 | 0.9  (0.1) | 0.14 | 123.4  (17.6) | 0.76 | 74.2  (8.7) | 0.6 | 47.1  (7.7) | 0.63 |
| E4+ | 7 | 0 | 1 | 0 | 27.92  (6.73) |  | 0.81  (0.2) |  | 126  (23.1) |  | 76.8  (15.9) |  | 48.9  (9.2) |  |

**Supplemental Table 3.** Clinical characteristics of cognitively unimpaired individuals according to Ε4 carriage and age cohort (young=18-39, middle-aged=40-65). Values represent means +/- (SD). Ca, Caucasian; AA, African American; His, Hispanic; A, Asian; BMI, body mass index.

| *(all comparisons shown are E4+ vs E4-)* | | Pre-glucose challenge | | Post-glucose challenge | |
| --- | --- | --- | --- | --- | --- |
| **Metabolite** | **HMDB ID** | **Ratio** | **FDR** | **Ratio** | **FDR** |
| beta-alanine | HMDB0000056 | 1.059 | 0.797 | 1.179 | 0.314 |
| cholesterol | HMDB0000067 | 1.176 | 0.541 | 1.084 | 0.699 |
| citrate | HMDB0000094 | 1.258 | 0.273 | 1.276 | 0.152 |
| GABA | HMDB0000112 | 1.203 | 0.532 | 1.419 | 0.096 |
| glyoxylic acid | HMDB0000119 | 0.822 | 0.489 | 0.891 | 0.542 |
| glycine | HMDB0000123 | 1.102 | 0.717 | 1.098 | 0.546 |
| glycerol 3-phosphate | HMDB0000126 | 1.002 | 0.994 | 0.853 | 0.542 |
| fumaric acid | HMDB0000134 | 0.940 | 0.797 | 0.822 | 0.314 |
| glyceric acid | HMDB0000139 | 1.243 | 0.193 | 1.310 | **0.034** |
| glutamic acid | HMDB0000148 | 1.427 | 0.066 | 1.757 | **0.011** |
| ethanolamine | HMDB0000149 | 1.141 | 0.532 | 1.160 | 0.291 |
| tyrosine | HMDB0000158 | 1.072 | 0.797 | 1.331 | 0.153 |
| phenylalanine | HMDB0000159 | 0.999 | 0.994 | 1.141 | 0.354 |
| maltose | HMDB0000163 | 1.143 | 0.775 | 1.681 | **0.028** |
| threonine | HMDB0000167 | 0.597 | 0.107 | 0.897 | 0.546 |
| isoleucine | HMDB0000172 | 1.063 | 0.798 | 1.183 | 0.299 |
| lysine | HMDB0000182 | 0.929 | 0.797 | 1.197 | 0.216 |
| lactose | HMDB0000186 | 1.218 | 0.466 | 1.471 | 0.057 |
| serine | HMDB0000187 | 1.025 | 0.919 | 1.277 | 0.118 |
| lactate | HMDB0000190 | 1.519 | **0.001** | 1.264 | **0.013** |
| oleic acid | HMDB0000207 | 0.722 | 0.294 | 0.991 | 0.957 |
| α-ketoglutarate | HMDB0000208 | 1.084 | 0.775 | 1.057 | 0.715 |
| myo-inositol | HMDB0000211 | 1.149 | 0.532 | 1.508 | **0.028** |
| n-acetylgalactosamine | HMDB0000212 | 1.507 | 0.054 | 1.359 | 0.275 |
| palmitic acid (polar lipids) | HMDB0000220 | 1.074 | 0.797 | 1.341 | 0.098 |
| n-acetyl-neuraminic acid | HMDB0000230 | 1.026 | 0.919 | 1.676 | **0.013** |
| pyruvate | HMDB0000243 | 1.128 | 0.532 | 1.108 | 0.464 |
| sucrose | HMDB0000258 | 0.817 | 0.649 | 0.791 | 0.326 |
| serotonin | HMDB0000259 | 0.757 | 0.242 | 1.032 | 0.861 |
| pyroglutamic acid | HMDB0000267 | 1.256 | 0.312 | 1.336 | 0.153 |
| ribose | HMDB0000283 | 0.9659 | 0.894 | 1.102 | 0.546 |
| urea | HMDB0000294 | 1.1415 | 0.621 | 1.117 | 0.441 |
| creatinine | HMDB0000562 | 0.7535 | 0.445 | 0.701 | 0.098 |
| fructose | HMDB0000660 | 1.212 | 0.489 | 1.429 | **0.028** |
| linoleic acid polar | HMDB0000673 | 0.914 | 0.797 | 1.066 | 0.750 |
| methionine | HMDB0000696 | 1.268 | 0.358 | 1.205 | 0.313 |
| homoserine | HMDB0000719 | 0.857 | 0.647 | 0.688 | 0.098 |
| malic acid | HMDB0000744 | 1.185 | 0.441 | 1.185 | 0.247 |
| 3-phosphoglyceric acid | HMDB0000807 | 1.306 | 0.0843 | 1.398 | **0.018** |
| stearic acid | HMDB0000827 | 1.156 | 0.532 | 1.317 | 0.143 |
| valine | HMDB0000883 | 1.174 | 0.533 | 1.217 | 0.284 |
| tryptophan | HMDB0000929 | 0.957 | 0.822 | 1.105 | 0.54 |
| threonic acid | HMDB0000943 | 1.464 | 0.065 | 1.977 | **0.0013** |
| F16BP | HMDB0001058 | 0.783 | 0.242 | 0.927 | 0.584 |
| glucosamine-1-phosphate | HMDB0001109 | 0.739 | 0.193 | 0.883 | 0.527 |
| alanine | HMDB0001310 | 1.018 | 0.919 | 1.192 | 0.244 |
| guanosine-5-phosphate | HMDB0001397 | 1.063 | 0.797 | 0.848 | 0.353 |
| glucosamine | HMDB0001514 | 1.531 | 0.066 | 1.724 | **0.002** |
| oxalic acid | HMDB0002329 | 1.024 | 0.919 | 0.929 | 0.715 |
| threose | HMDB0002649 | 0.772 | 0.414 | 1.043 | 0.772 |
| n-acetyl-tryptophan | HMDB0013713 | 1.076 | 0.797 | 1.220 | 0.354 |
| acetohydroxamic acid | HMDB0014691 | 1.186 | 0.532 | 1.187 | 0.353 |
| aspartyl-glutamate | HMDB0028752 | 0.581 | 0.162 | 0.576 | 0.096 |
| leucine | HMDB00687 | 1.077 | 0.797 | 1.265 | 0.152 |

**Supplemental Table 4.** Plasma metabolites of study participants analyzed by gas chromatography – before and after a dietary glucose challenge.

| Pre-Existing Symptoms Checklist | **INSTRUCTIONS:** Review this list at each visit. If any symptom is present at Baseline, be sure to report it on the ***on medical history*** | |  |
| --- | --- | --- | --- |
| **Symptom** | Yes No | Comments | |
| **1.** pregnant or breastfeeding | Yes No |  | |
| **2.** have a bleeding disorder | Yes No |  | |
| **3.** allergy to the local anesthetic lidocaine | Yes No |  | |
| **4.** history of stroke, seizures, Parkinson’s disease, history of head injury with loss of consciousness, or other dementing disorder | Yes No |  | |
| **5.** history of alcoholism or drug abuse | Yes No |  | |
| **6.** History of schizophrenia or currently suffer from bipolar disorder or major depression. | Yes No |  | |
| **7.** vision or hearing loss severe enough to interfere with cognitive testing | Yes No |  | |
| **8.** Taking Beta Blockers (ex. Sectral, Tenormin, Zebeta, Lopressor, Corgard, Bystolic, Inderal LA, InnoPran XL) | Yes No |  | |
| **9.** Taking Neuroleptics (ex. Clozaril, Saphris, Zeprexa, Seroquel) | Yes No |  | |
| **10.** Taking Narcotic Analgesics (ex. Codeine, Zohydro ER, Oxycodone, Methadone, Hydromorphone, Morphine, Fentanyl) | Yes No |  | |
| **11.** Taking Anti-Parkinsonian Agents (ex. Sinemet, Symmetrel, Artane, Cogentin, Elderpryl, Azliect, Comtan) | Yes No |  | |
| **12.** Taking CNS-Active antihypertensive agents (ex. Catapres, Kapvay, Intuniv, Tenex) | Yes No |  | |
| **Information obtained by:** |  |  | |

**Supplemental Table 5.** Pre-screening checklist. A response of “yes” to any of the following resulted in exclusion from the study.


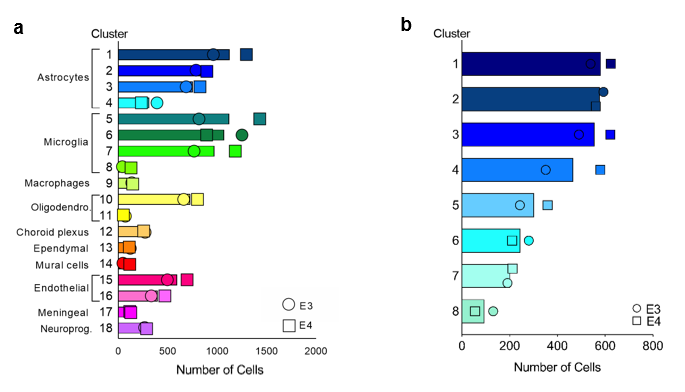


**Supplemental Fig. 1 Cluster cell counts.** Number of cells in each graph-based cluster from all cells **(a)**, and astrocytes only **(b)**. Bars represent mean number of cells in each cluster, with the number of E3 cells (circles) and E4 cells (squares) noted by symbols.


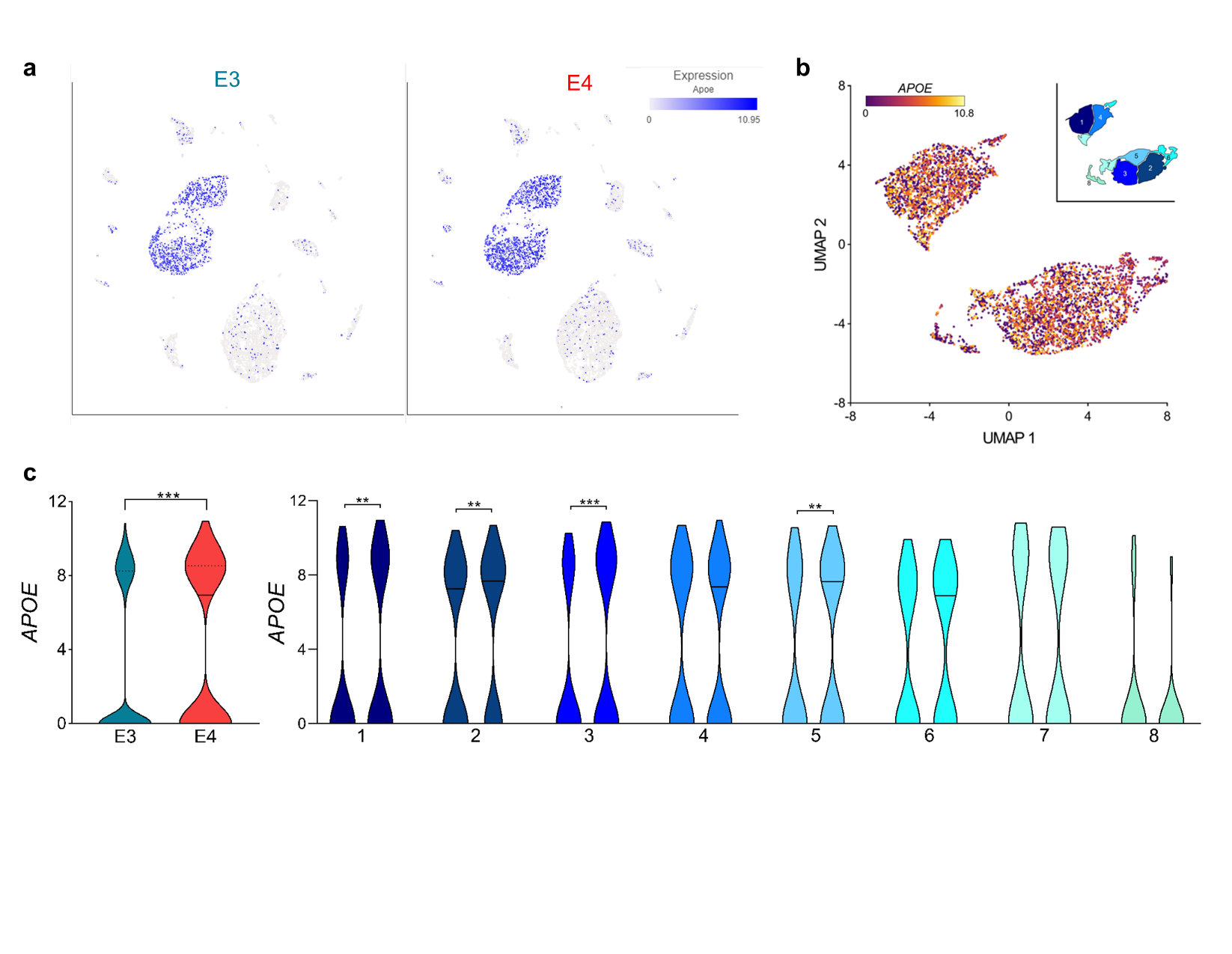


**Supplemental Fig. 2 *APOE* expression in single-cells and specific astrocyte clusters.** (a) UMAP visualization of E3 (left) and E4 (right) cells showing expression of *APOE.* *APOE* expression is primarily limited to cells identified as astrocytes. (b) Expression of *APOE* in astrocyte-only UMAP (*Aldoc+* cells). Inset shows the 8 distinct astrocyte clusters. (c) Violin plots showing expression of *APOE* in all astrocytes (left) and within each astrocyte cluster (right). (***P*<0.01, ***P<0.001, unpaired *t*-test, two-tailed)


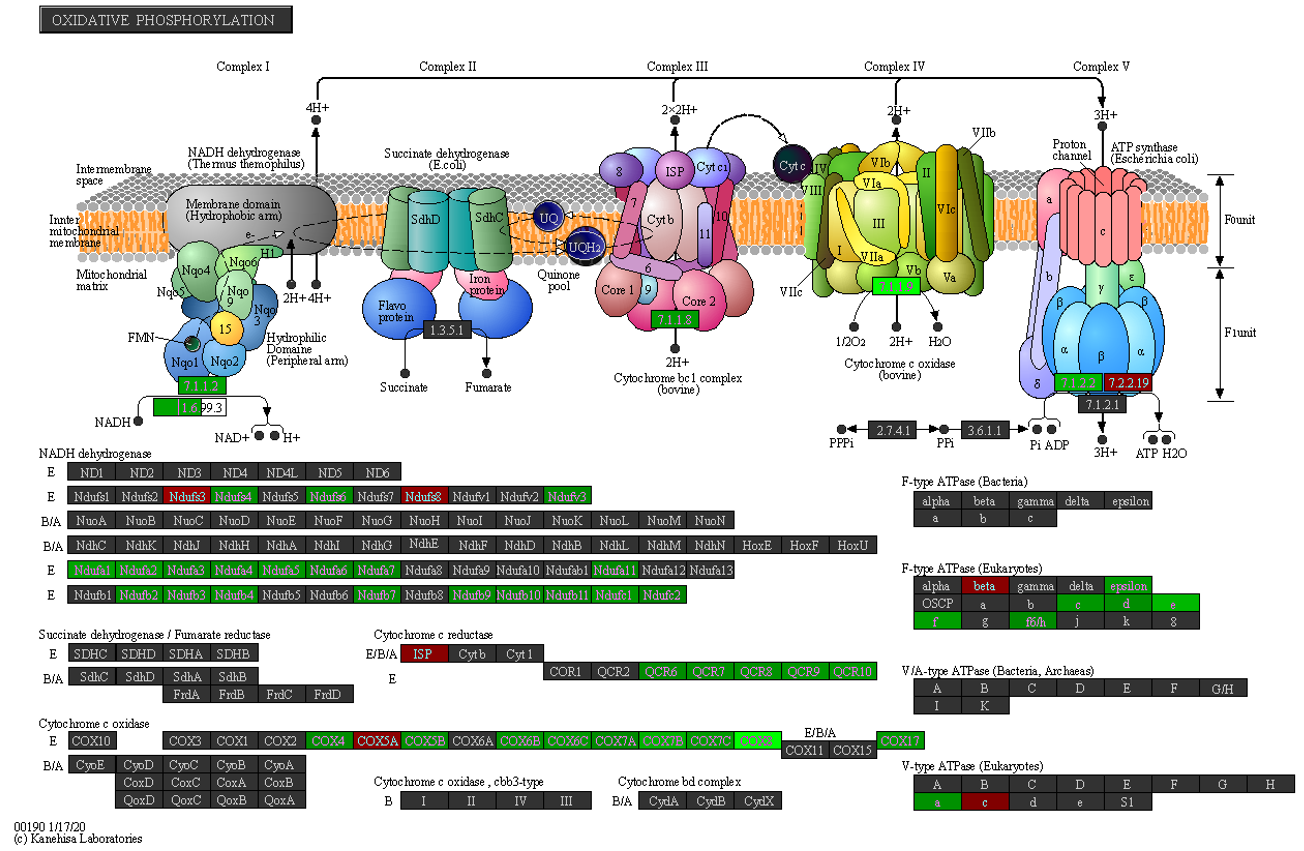

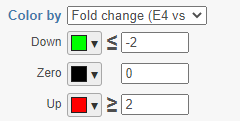


**Supplemental Fig. 3 E4 is associated with decreases in many genes of the oxidative phosphorylation KEGG pathway.** Pathway map for KEGG pathway “Oxidative Phosphorylation” showing genes differentially expressed between E3 and E4 astrocytes. Genes highlighted in green are downregulated in E4, genes in red are upregulated in E4.


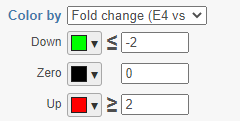
**
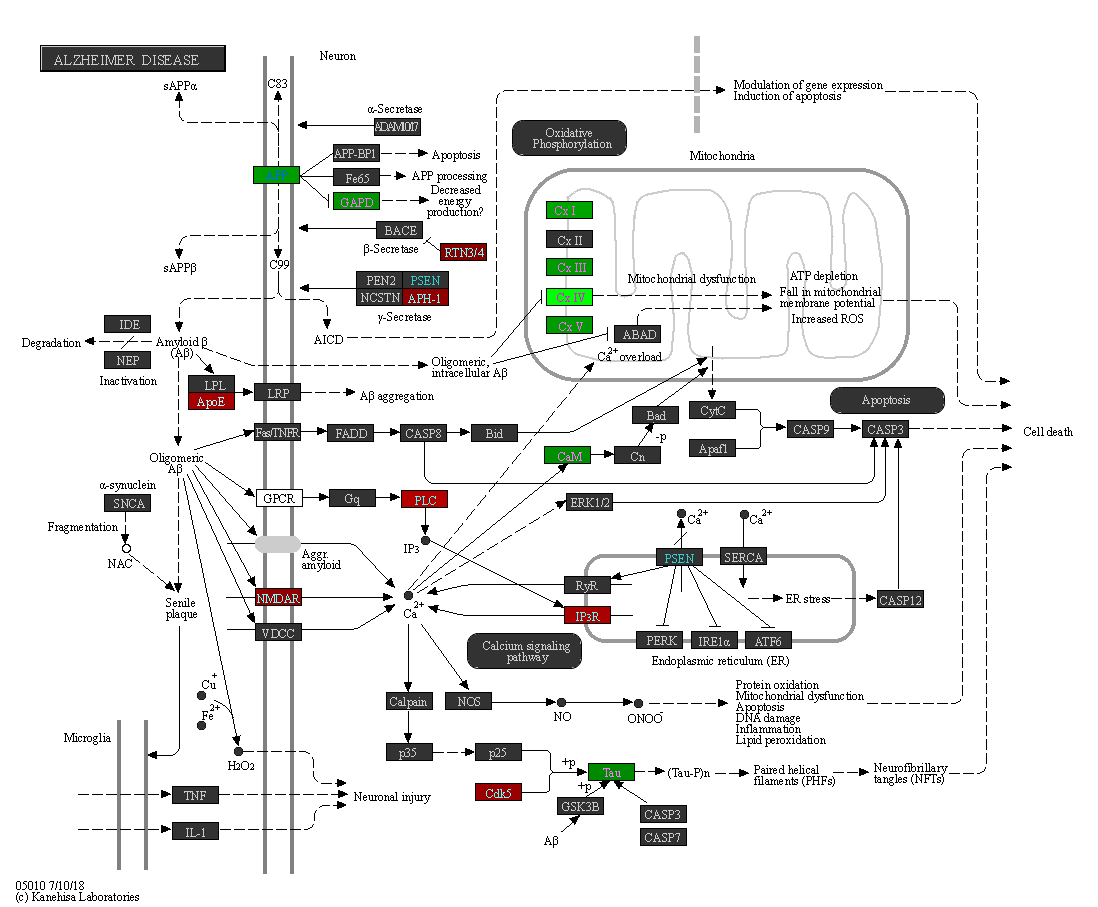
**

**Supplemental Fig. 4 E4 is associated with decreases in many genes of the “Alzheimer’s disease” KEGG pathway.** Pathway map for KEGG pathway “Oxidative Phosphorylation” showing genes differentially expressed between E3 and E4 astrocytes. Genes highlighted in green are downregulated in E4, genes in red are upregulated in E4.


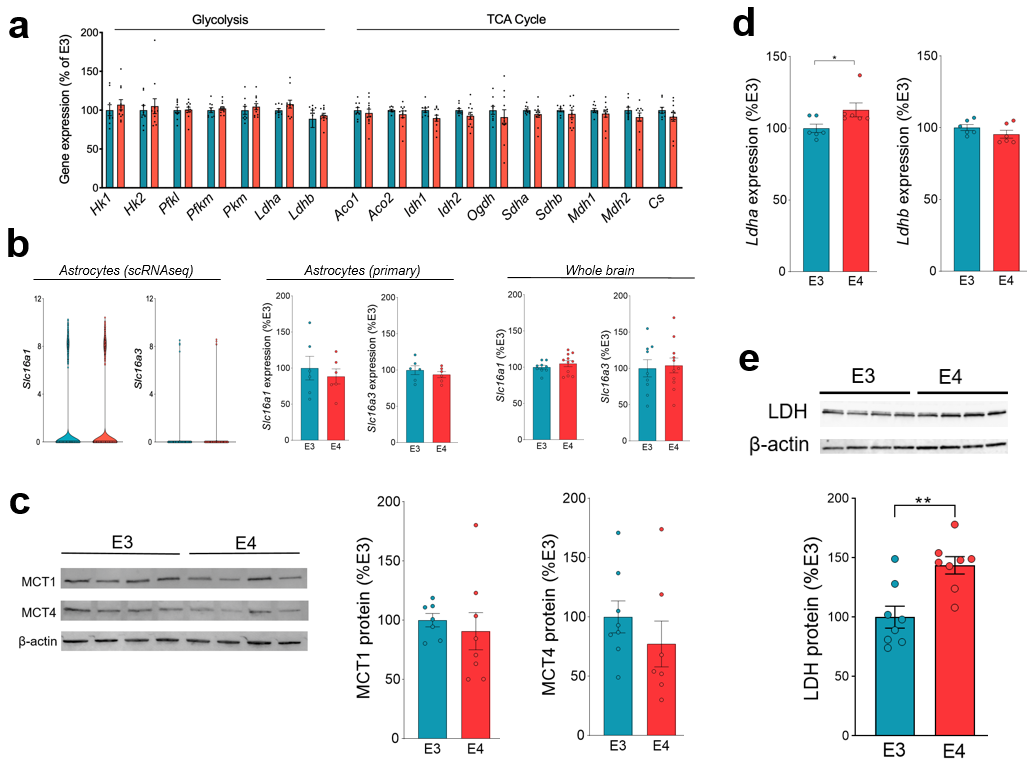


**Supplemental Fig. 5 LDH expression is increased in E4 astrocytes; MCT expression is unchanged. (a)** Gene expression of critical enzymes in glycolysis and TCA cycle in whole brain homogenates from female E3 and E4 mice. *Hk*, hexokinase; *Pfk*, phosphofructokinase; *Ldh*, lactate dehydrogenase; *Aco*, aconitase; *Idh*, isocitrate dehydrogenase; *Ogdh*, oxoglutarate dehydrogenase; *Sdh*, succinate dehydrogenase; *Mdh*, malate dehydrogenase; *Cs*, citrate synthase. Data analyzed by multiple t-tests with Sidak multiple comparison correction. **(b)** *Slc16a1* and *Slc16a3* gene expression in astrocytes from the scRNAseq data from Figure 1 (*left*), in primary astrocytes isolated from E3 or E4 mice (*middle*), and in whole brain homogenates from female E3 or E4 mice (*right*). **(c)** MCT1 and MCT4 expression was quantified in brain tissue from mice expressing E3 or E4 via western blot (n=7-8). MCT expression normalized to β-actin loading control and expressed as a percent of E3 (value/mean E3). **(d)** *Ldha* and *Ldhb* gene expression in primary astrocytes. All gene expression values expressed as a percent of E3 (value/mean E3). ******P*<0.05, ***P*<0.01, t-test. **(e)** LDH protein expression was measured via western blot in primary astrocytes expressing E3 or E4 (n=8). LDH expression normalized to β-actin loading control and expressed as a percent of E3 (value/mean E3). *****p<0.05, t-test.


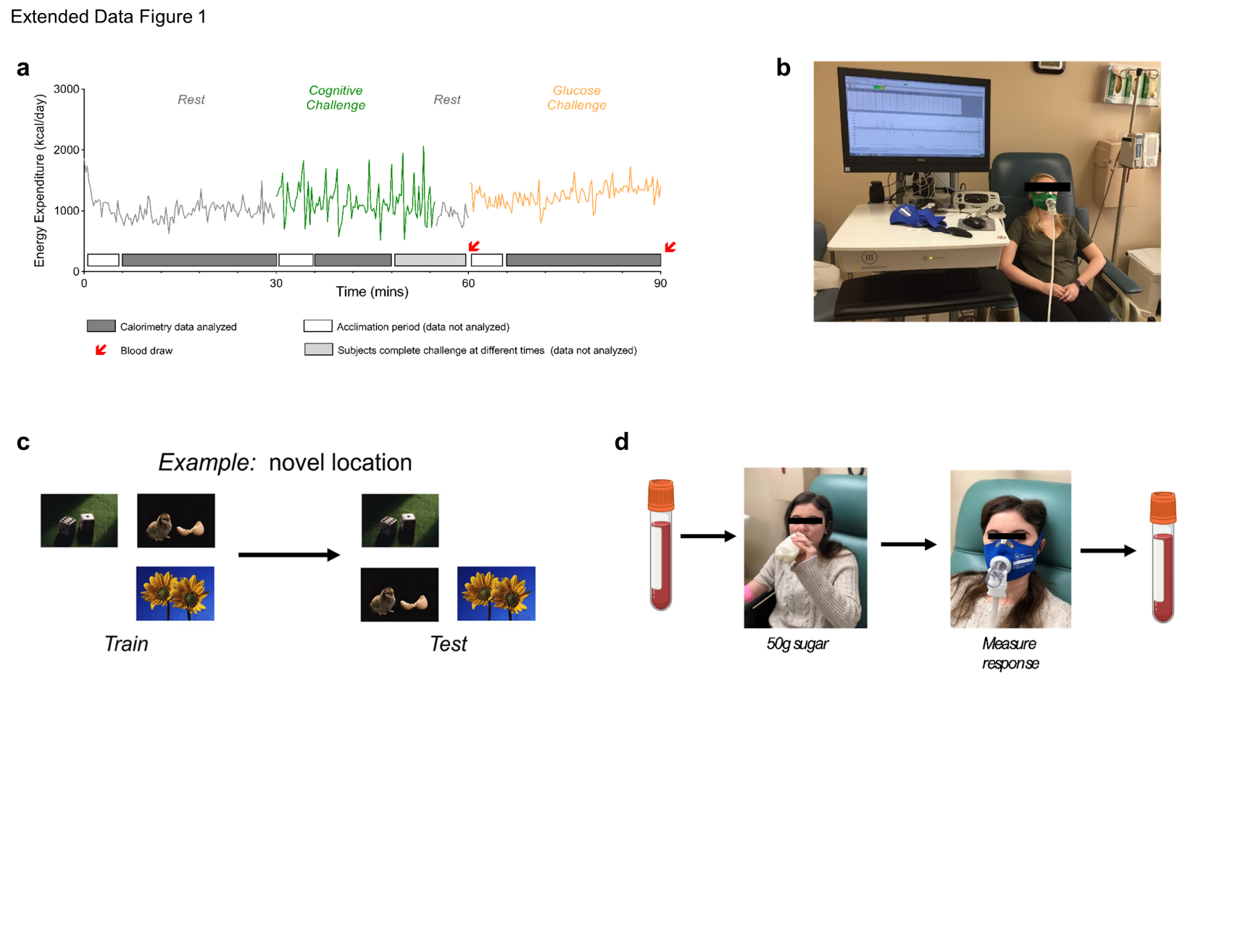


**Supplemental Fig. 6 Human indirect calorimetry study design. (a)** Representative time course of energy expenditure (EE) measures during the three periods of the study (rest in gray, cognitive challenge in green, and glucose challenge in orange). Data was only analyzed during the last 25 minutes of the resting and glucose periods and during a common 5-15 minute span during the cognitive challenge in which all 100 subjects were actively engaged in the task – denoted by grey bar on x axis. Blood was drawn immediately prior and after the glucose challenge. **(b)** Representative photo of a participant during the resting challenge connected to the Ultima MGX indirect calorimetry (IC) system. **(c)** Example slides from the Novel Image Novel Location test used as a cognitive challenge. **(d)** The glucose challenge consisted of a blood draw, followed by ingestion of the 50g sugar drink (all subjects consumed drink within 90 seconds), followed by IC measurement, and a second blood draw.


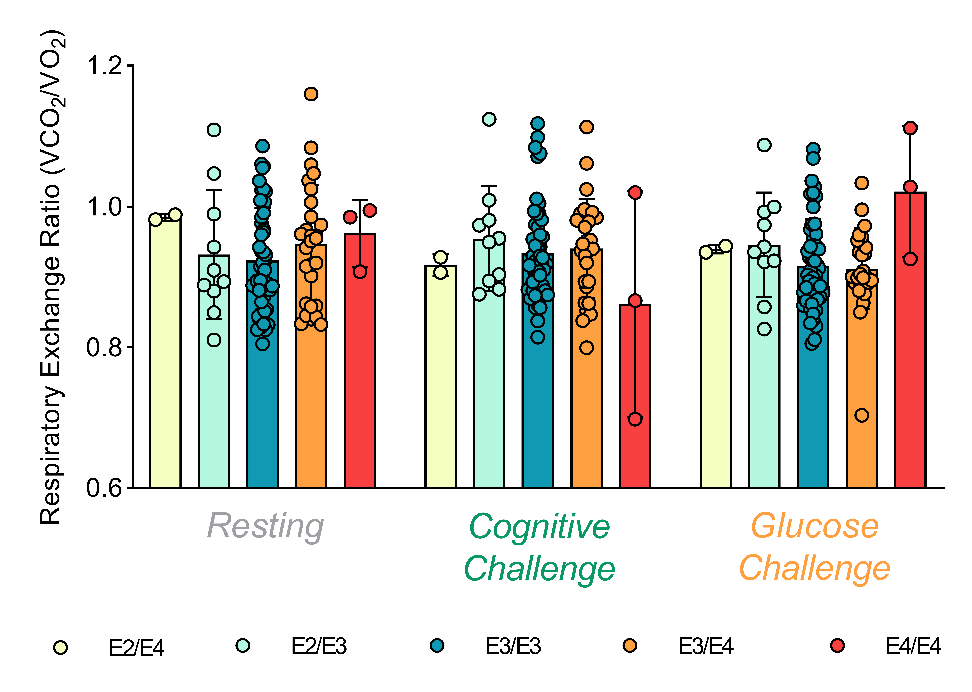


**Supplemental Fig. 7 Respiratory Exchange Ratio (RER) does not differ by *APOE* genotype.** Respiratory exchange ratio (RER) (VCO_2_/VO_2_) was not significantly different between *APOE* genotypes across any of the three periods tested.


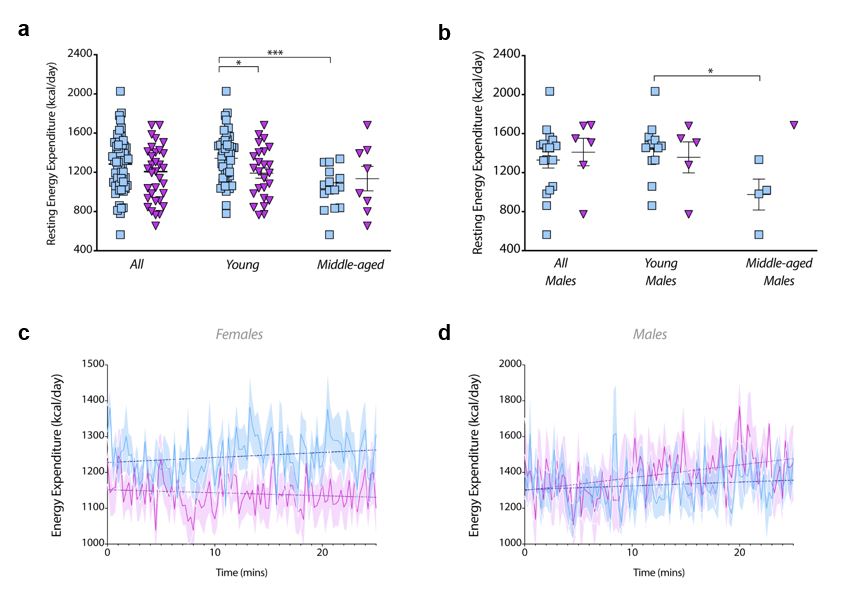


**Supplemental Fig. 8 E4 effect on resting energy expenditure (a)** E4 non-carriers’ (n=61; blue) and E4 carriers’ (n=33; purple) average resting energy expenditures were determined and stratified by young and middle-aged. (*P<0.05, ***P<0.001, unpaired t-test, two-tailed). **(b)** This was repeated for only male participants (*P<0.05, unpaired t-test, two-tailed; E4- total n=17, young n=13, middle-aged n=4; E4+ total n=6, young n=5, middle-aged n=1). **(c)** Average EE was plotted over the resting period for females and **(d)** males. Dotted lines indicate liner regression results and shaded area are SEMs*.*

**
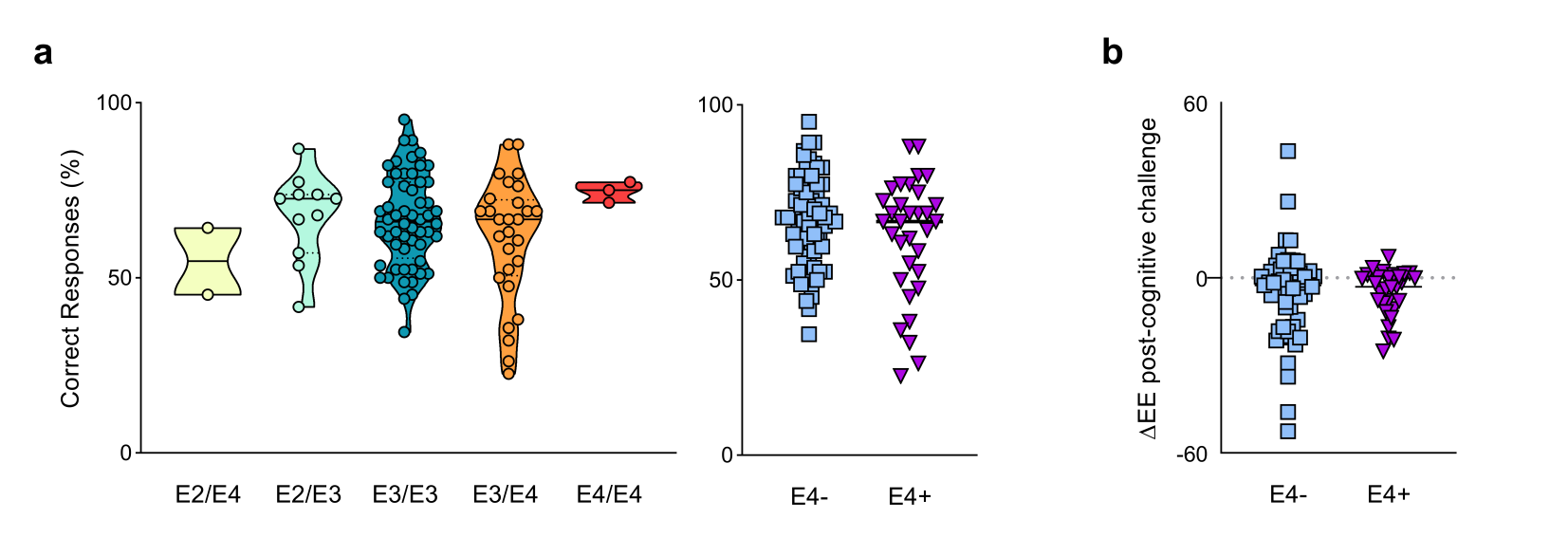
**

**Supplemental Fig. 9 Novel image novel location object recognition test response accuracy by APOE genotype. (a)** The novel-image-novel-location (NINL) object recognition test contains 7 sets of 12 slides. Each slide has 3 images and 4 possible locations. Each slide is viewed for eight seconds in the order as follows: See Set A, See Set B. Test Set A, See Set C, Test Set B, See Set D, Test Set C, etc. To be considered correct, subjects must identify both the type of change and in which quadrant the change has occurred. The test is designed so that on average subjects answer 60-80% of questions correctly. Total percent correct was calculated for each genotype **(b)** and stratified by E4 carriage. **(c)** Individual slopes of EE after the cognitive challenge showing an average decrease in EE after the challenge.

**
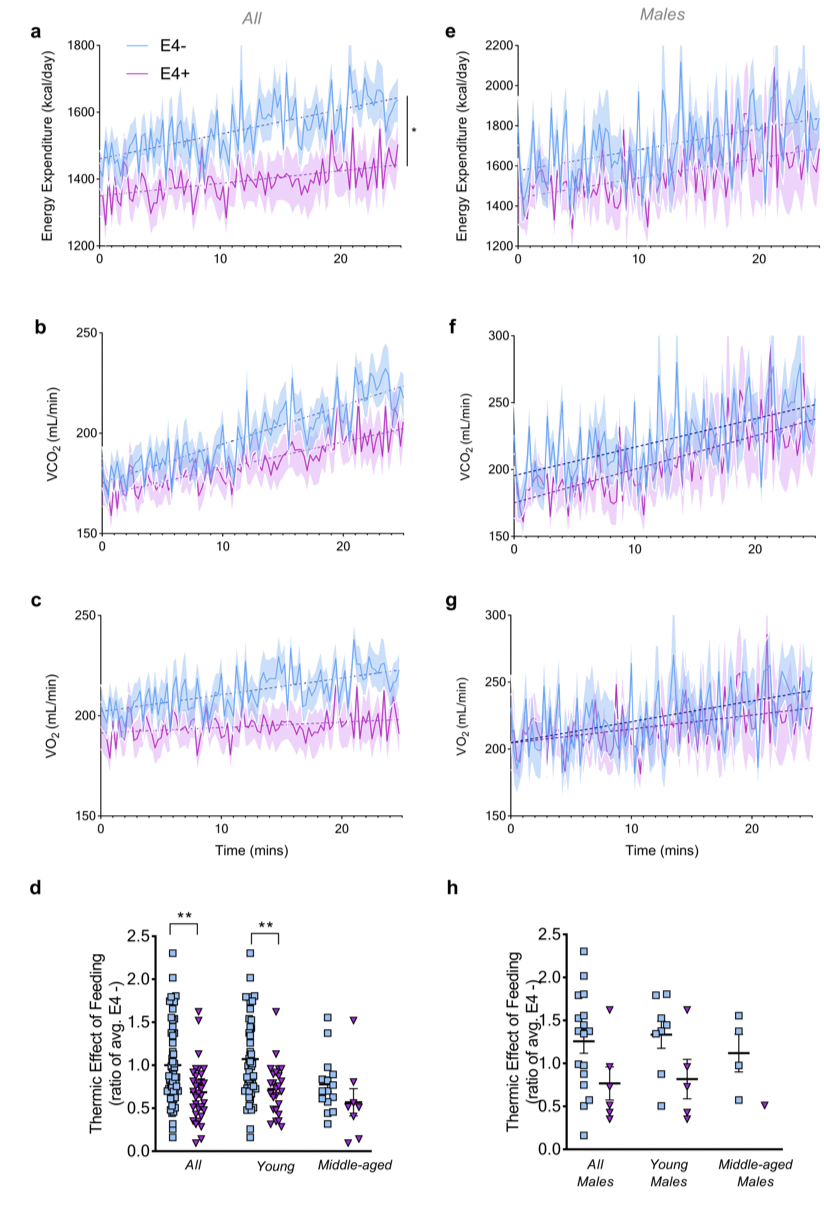
**

**Supplemental Fig. 10. E4 effect on energy expenditure during glucose challenge in all subjects (left column), and in males only (right column) (a)** Energy expenditure **(b)** VCO_2_ and **(c)** VO_2_ was plotted over the glucose challenge period in all E4- (n=61; blue) and E4+ (n=33; purple) participants. (*P<0.05, Two-way ANOVA repeated measures). **(d)** Thermic effect of feeding was determined as a ratio of E4 non-carriers in all, young, and middle-aged participants. (**P<0.01, unpaired t-test, two-tailed) **(e)** Energy expenditure **(f)** VCO_2_ and **(g)** VO_2_ was plotted over the glucose challenge period in male participants (E4- n=17; E4+ n=6). Dotted lines show linear regression trend line, shaded areas refer to SEM. **(h)** Thermic effect of feeding was determined as a ratio of E4 non-carriers in all, young, and middle-aged male participants.


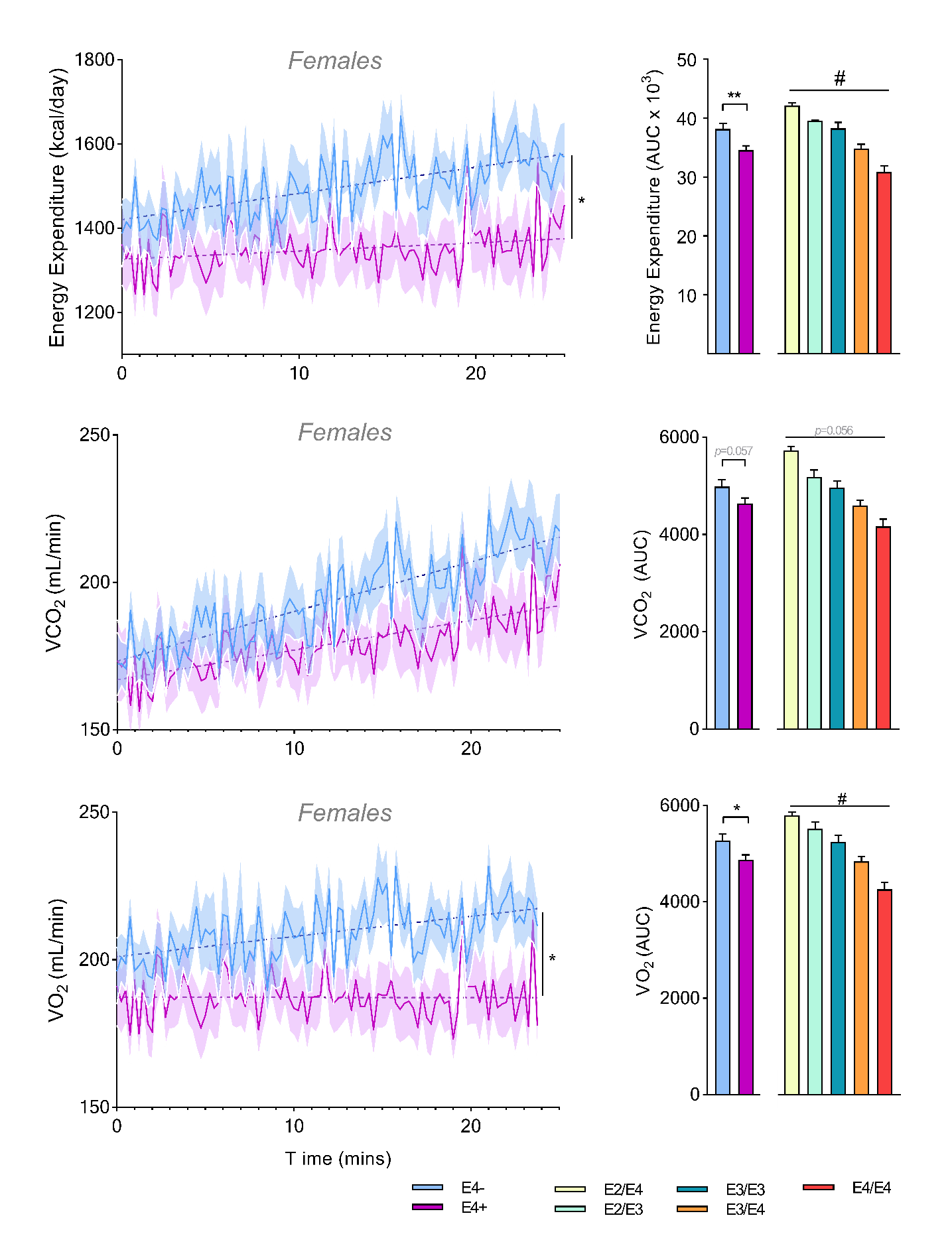

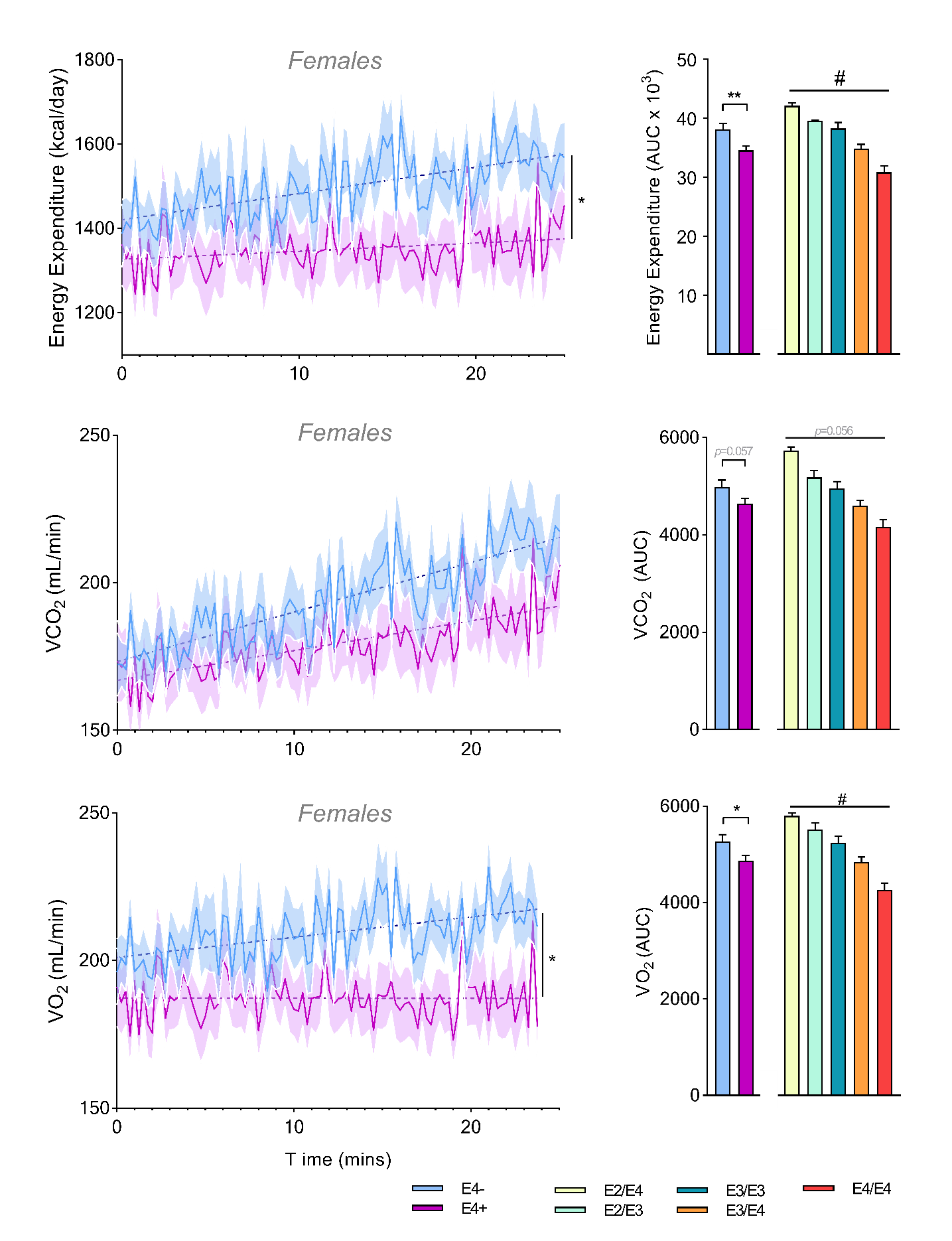


**Supplemental Fig. 11 VCO_2_ values during the glucose challenge period. (a)** Time course of average VCO2 values of Ε4- and Ε4+ females during the glucose challenge period. Dashed lines refer to linear regression result. **(b)** AUC of VCO_2_ for all participants. (a, Two-way ANOVA repeated measures; b, One-way ANOVA)


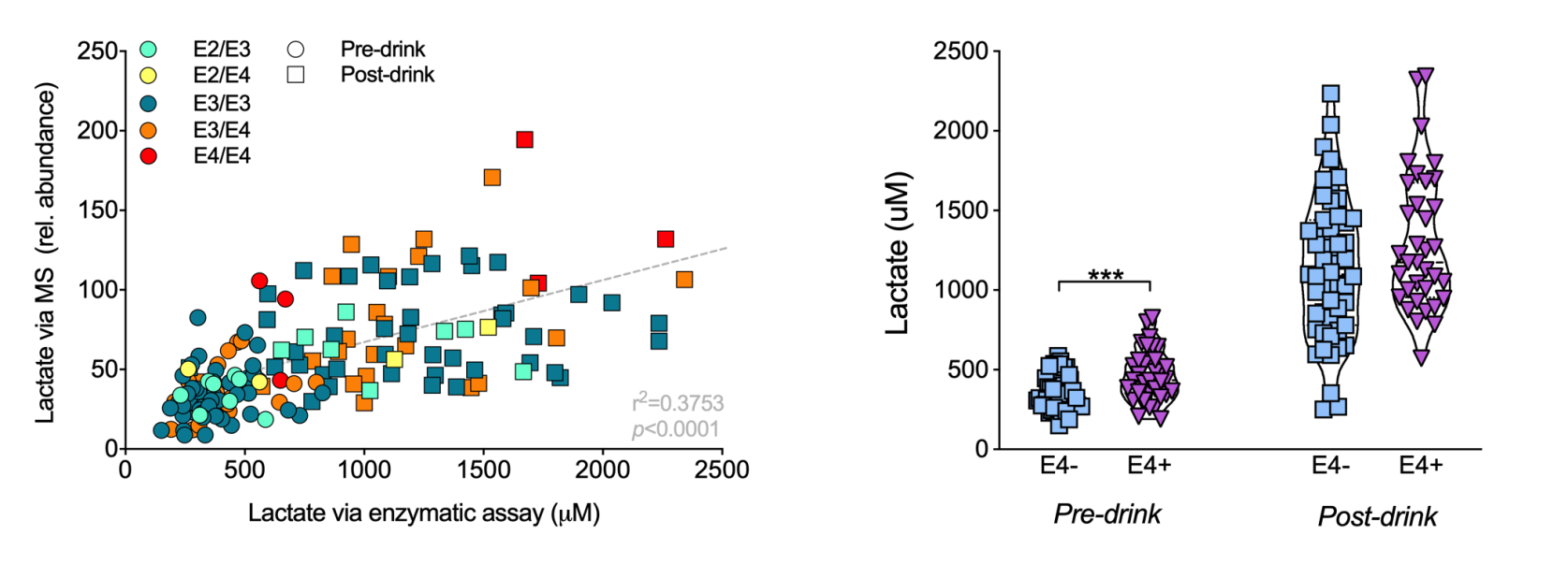


**Supplemental Fig. 12 Plasma lactate assessed via enzymatic assay. (a)** Lactate values quantified by GCMS (relative abundance, y-axis) strongly correlate with lactate values (uM) assessed via enzymatic assay. **(b)** E4 carriers had higher plasma lactate pre-drink and a trend toward higher lactate post-drink (p=0.09) compared to non-carriers, as measured via enzymatic assay.

**
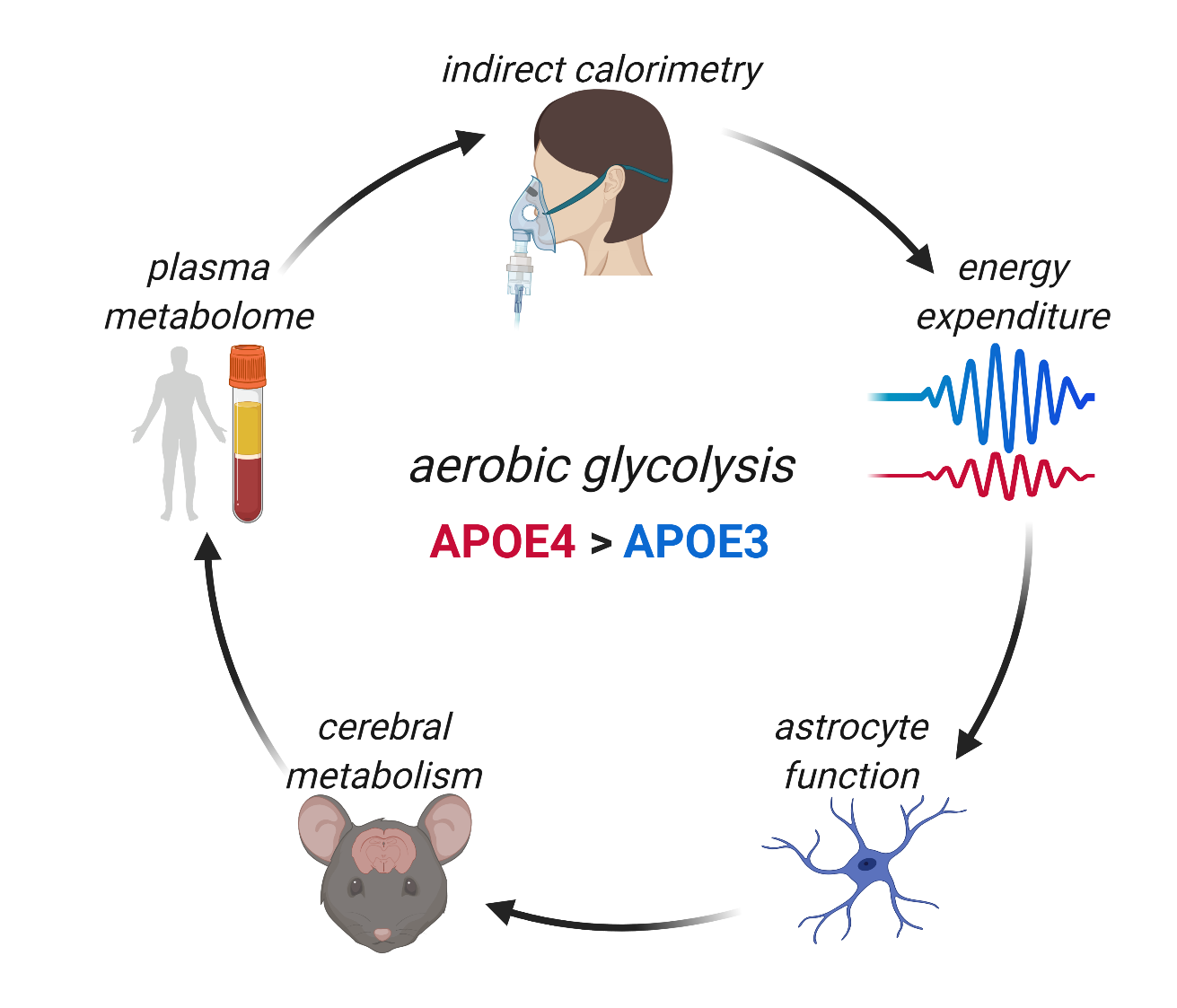
**

**Summary Figure**
